# Supplementary material for: Comparison of Two DNA Labeling Dyes Commonly Used to Detect Metabolically Active Bacteria
Source: Microorganisms. 2025 Apr 28;13(5):1015. doi: 10.3390/microorganisms13051015 (PMC12114394; doi:10.3390/microorganisms13051015)
Supplement: Supplementary file 1 [file microorganisms-13-01015-s001.zip › Malayil et_al_Supplementary Figures with legends.pdf]

Supplementary Figures with legends

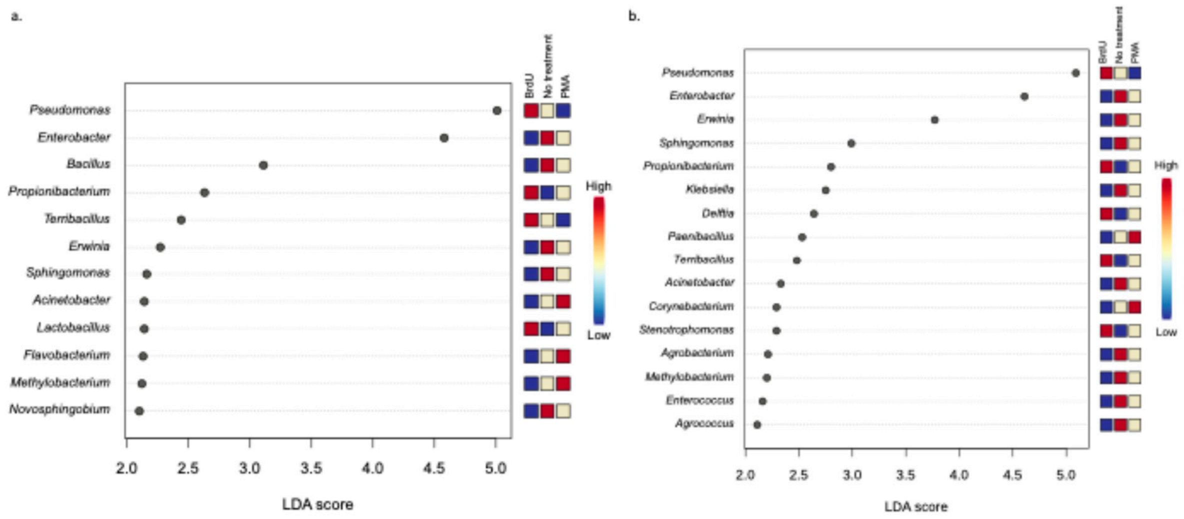

Supplementary Figure S1: LEfSe analysis on the differential abundance of bacteria between treatments (BrdU, no treatment and PMA) in manufactured samples: a) hookah and b) little cigars.

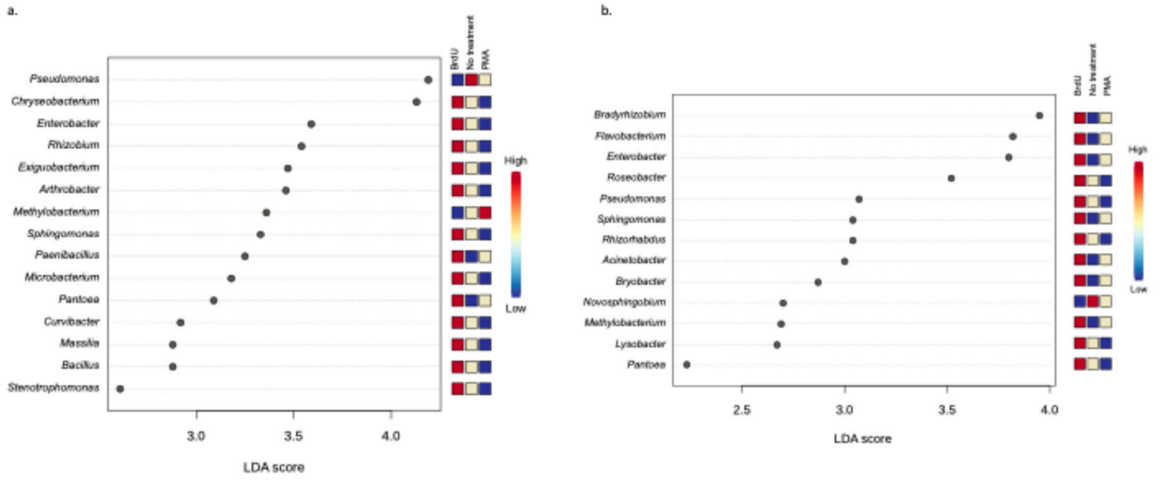

Supplementary Figure S2: LEfSe analysis on the differential abundance of bacteria between treatments (BrdU, no treatment and PMA) in natural samples: a) water and b) produce.

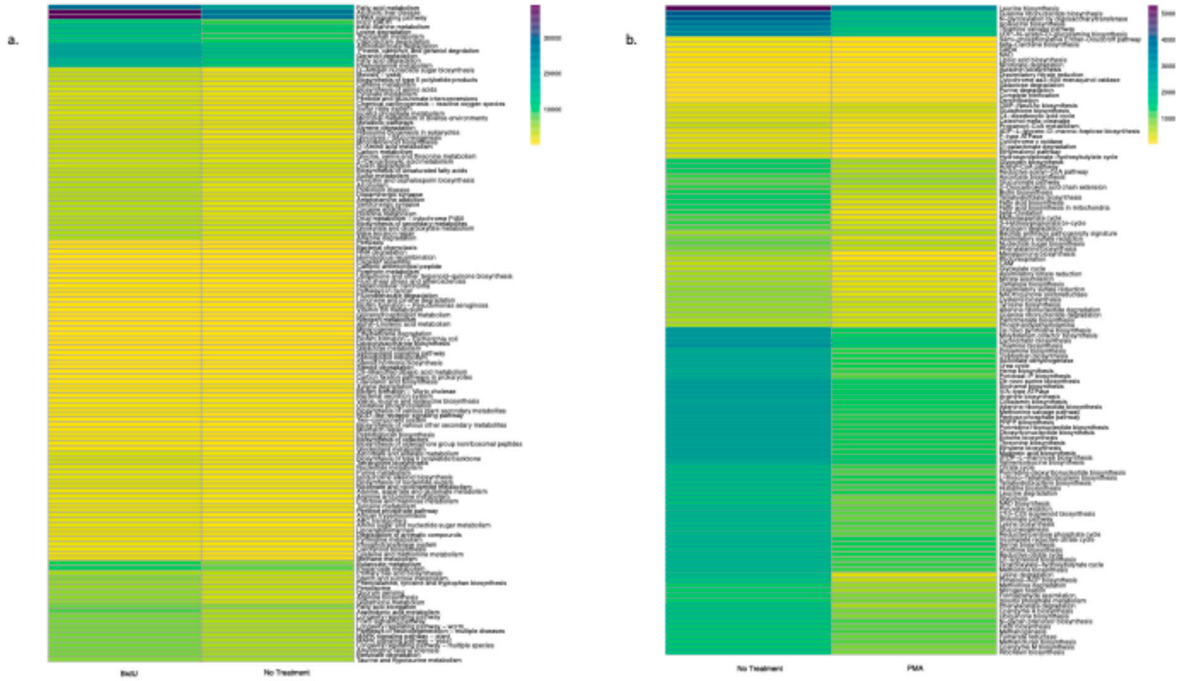

Supplementary Figure S3**.** Heatmap illustrating the functional profile predicted at level 2 KEGGs Orthologs using PICRUSt analysis of a natural sample (produce): a) BrdU-treated versus no treatment; and b) PMA-treated versus no treatment.

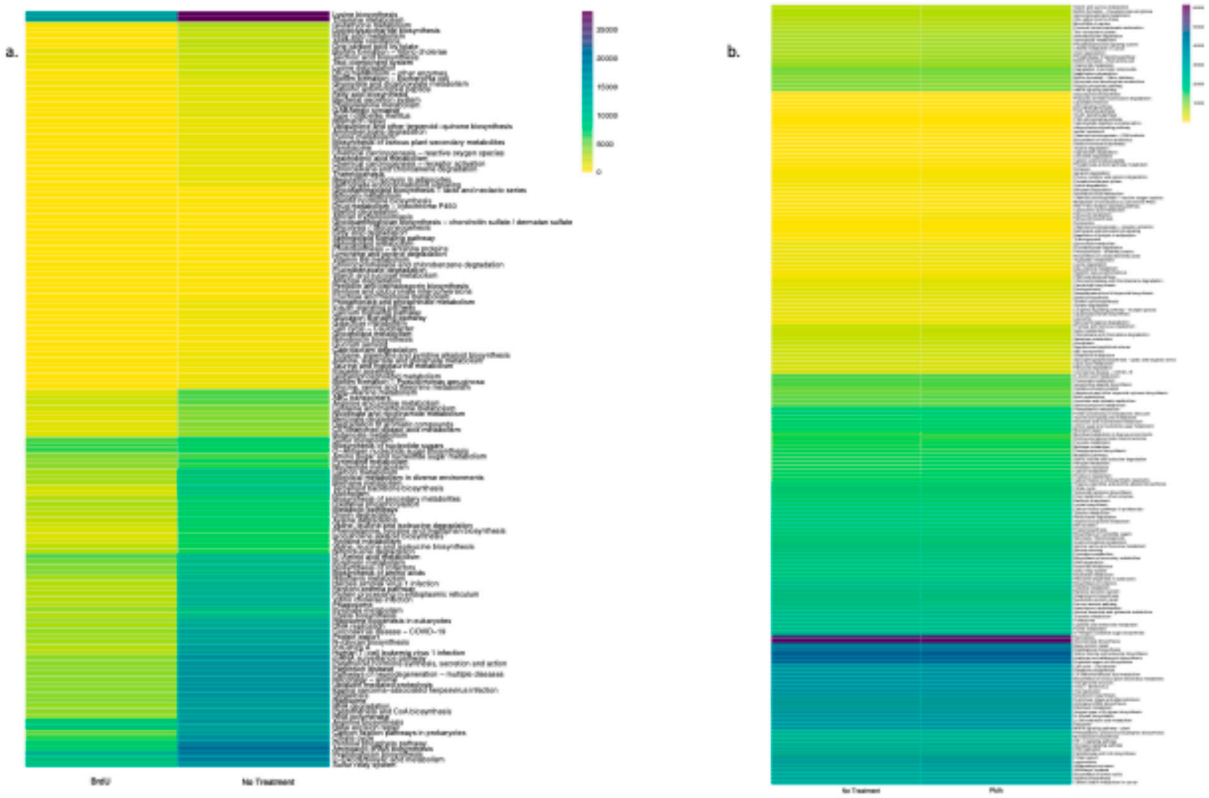

Supplementary Figure S4**.** Heatmap illustrating the functional profile predicted at level 2 KEGGs Orthologs using PICRUSt analysis of a natural sample (soil): a) BrdU-treated versus no treatment; and b) PMA-treated versus no treatment.

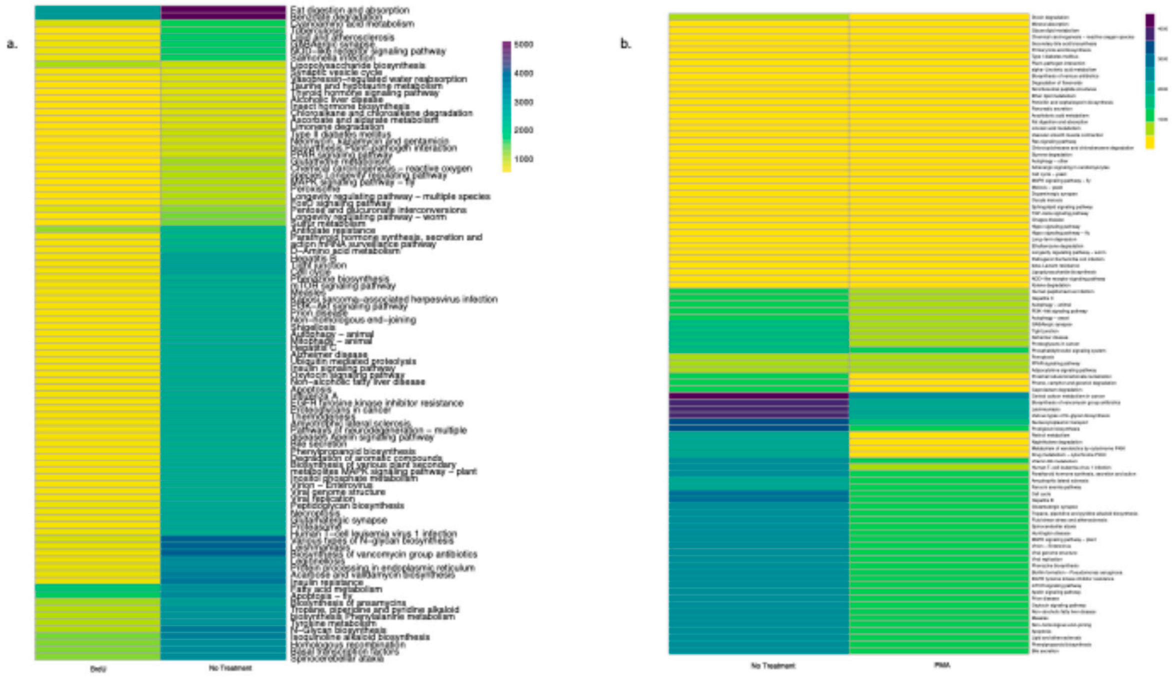

Supplementary Figure S5**.** Heatmap illustrating the functional profile predicted at level 2 KEGGs Orthologs using PICRUSt analysis of a manufactured sample (little cigar): a) BrdU-treated versus no treatment; and b) PMA-treated versus no treatment.

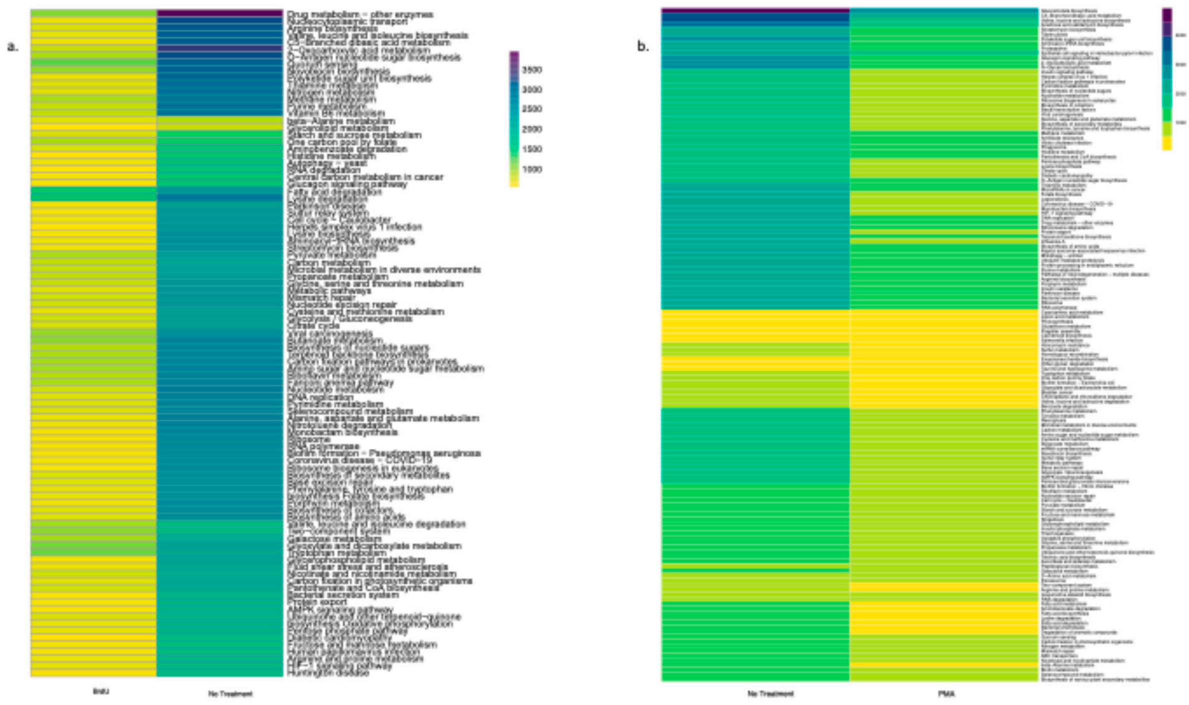

Supplementary Figure S6**.** Heatmap illustrating the functional profile predicted at level 2 KEGGs Orthologs using PICRUSt analysis of a manufactured sample (cigarettes): a) BrdU-treated versus no treatment; and b) PMA-treated versus no treatment.
