# Supplementary material for: Comparison of Two DNA Labeling Dyes Commonly Used to Detect Metabolically Active Bacteria
Source: Microorganisms. 2025 Apr 28;13(5):1015. doi: 10.3390/microorganisms13051015 (PMC12114394; doi:10.3390/microorganisms13051015)
Supplement: Supplementary file 1 [file microorganisms-13-01015-s001.zip › Supplementary Figure S3.pdf]

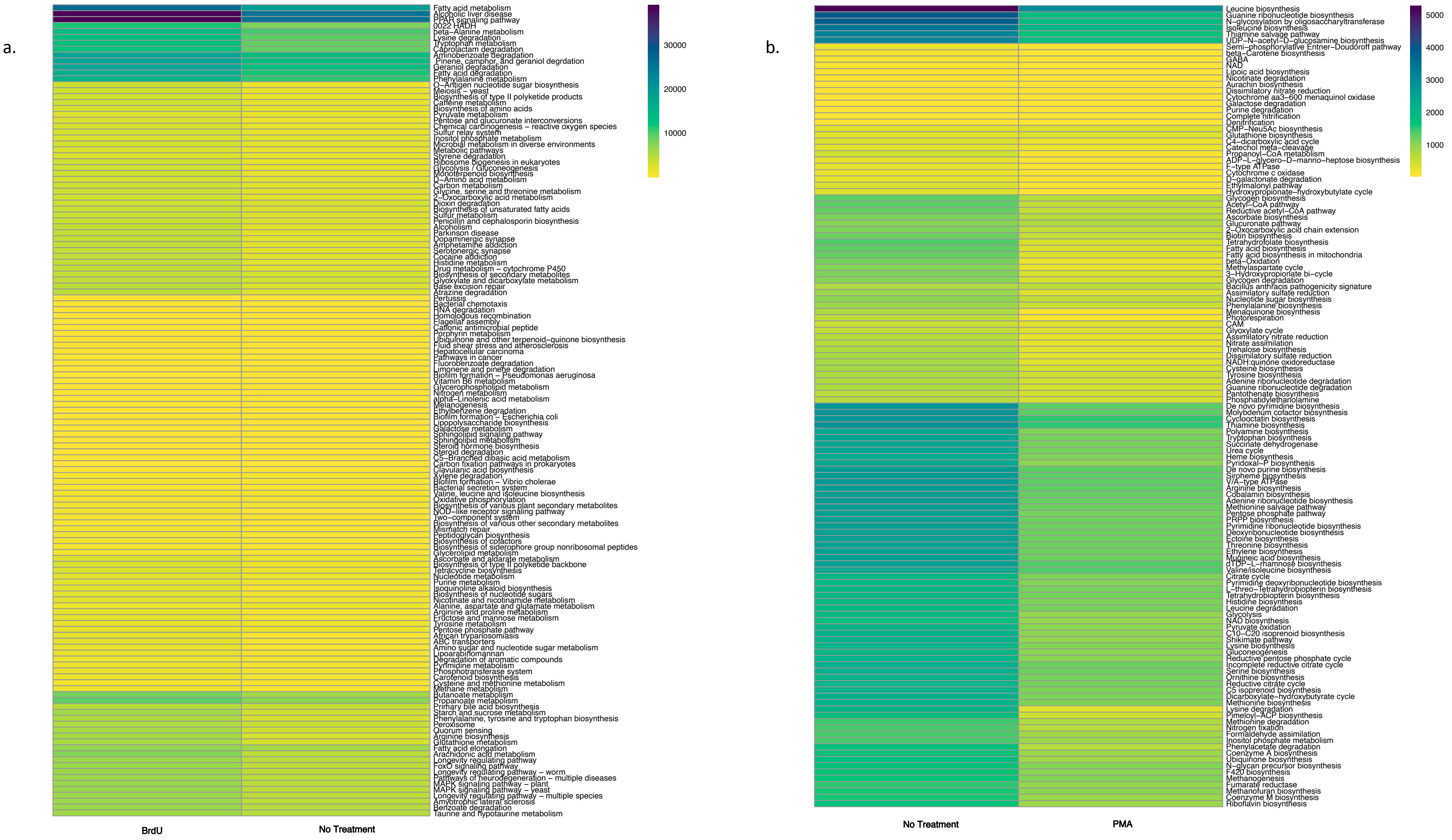
a.
Fatty acid metabolism
Alcoholic liver disease
PPAR signaling pathway
0022 HADH
beta−Alanine metabolism
Lysine degradation
Tryptophan metabolism
Caprolactam degradation
Aminobenzoate degradation
Pinene, camphor, and geraniol degrdation
Geraniol degradation
Fatty acid degradation
Phenylalanine metabolism
O−Antigen nucleotide sugar biosynthesis
Meiosis − yeast
Biosynthesis of type II polyketide products
Caffeine metabolism
Biosynthesis of amino acids
Pyruvate metabolism
Pentose and glucuronate interconversions
Chemical carcinogenesis − reactive oxygen species
Sulfur relay system
Inositol phosphate metabolism
Microbial metabolism in diverse environments
Metabolic pathways
Styrene degradation
Ribosome biogenesis in eukaryotes
Glycolysis / Gluconeogenesis
Monoterpenoid biosynthesis
D−Amino acid metabolism
Carbon metabolism
Glycine, serine and threonine metabolism
2−Oxocarboxylic acid metabolism
Dioxin degradation
Biosynthesis of unsaturated fatty acids
Sulfur metabolism
Penicillin and cephalosporin biosynthesis
Alcoholism
Parkinson disease
Dopaminergic synapse
Amphetamine addiction
Serotonergic synapse
Cocaine addiction
Histidine metabolism
Drug metabolism − cytochrome P450
Biosynthesis of secondary metabolites
Glyoxylate and dicarboxylate metabolism
Base excision repair
Atrazine degradation
Pertussis
Bacterial chemotaxis
RNA degradation
Homologous recombination
Flagellar assembly
Cationic antimicrobial peptide
Porphyrin metabolism
Ubiquinone and other terpenoid−quinone biosynthesis
Fluid shear stress and atherosclerosis
Hepatocellular carcinoma
Pathways in cancer
Fluorobenzoate degradation
Limonene and pinene degradation
Biofilm formation − Pseudomonas aeruginosa
Vitamin B6 metabolism
Glycerophospholipid metabolism
Nitrogen metabolism
alpha−Linolenic acid metabolism
Melanogenesis
Ethylbenzene degradation
Biofilm formation − Escherichia coli
Lipopolysaccharide biosynthesis
Galactose metabolism
Sphingolipid signaling pathway
Sphingolipid metabolism
Steroid hormone biosynthesis
Steroid degradation
C5−Branched dibasic acid metabolism
Carbon fixation pathways in prokaryotes
Clavulanic acid biosynthesis
Xylene degradation
Biofilm formation − Vibrio cholerae
Bacterial secretion system
Valine, leucine and isoleucine biosynthesis
Oxidative phosphorylation
Biosynthesis of various plant secondary metabolites
NOD−like receptor signaling pathway
Two−component system
Biosynthesis of various other secondary metabolites
Mismatch repair
Peptidoglycan biosynthesis
Biosynthesis of cofactors
Biosynthesis of siderophore group nonribosomal peptides
Glycerolipid metabolism
Ascorbate and aldarate metabolism
Biosynthesis of type II polyketide backbone
Tetracycline biosynthesis
Nucleotide metabolism
Purine metabolism
Isoquinoline alkaloid biosynthesis
Biosynthesis of nucleotide sugars
Nicotinate and nicotinamide metabolism
Alanine, aspartate and glutamate metabolism
Arginine and proline metabolism
Fructose and mannose metabolism
Tyrosine metabolism
Pentose phosphate pathway
African trypanosomiasis
ABC transporters
Amino sugar and nucleotide sugar metabolism
Lipoarabinomannan
Degradation of aromatic compounds
Pyrimidine metabolism
Phosphotransferase system
Carotenoid biosynthesis
Cysteine and methionine metabolism
Methane metabolism
Butanoate metabolism
Propanoate metabolism
Primary bile acid biosynthesis
Starch and sucrose metabolism
Phenylalanine, tyrosine and tryptophan biosynthesis
Peroxisome
Quorum sensing
Arginine biosynthesis
Glutathione metabolism
Fatty acid elongation
Arachidonic acid metabolism
Longevity regulating pathway
FoxO signaling pathway
Longevity regulating pathway − worm
Pathways of neurodegeneration − multiple diseases
MAPK signaling pathway − plant
MAPK signaling pathway − yeast
Longevity regulating pathway − multiple species
Amyotrophic lateral sclerosis
Benzoate degradation
Taurine and hypotaurine metabolism
BrdU
No Treatment
30000
20000
10000
b.
Leucine biosynthesis
Guanine ribonucleotide biosynthesis
N−glycosylation by oligosaccharyltransferase
Isoleucine biosynthesis
Thiamine salvage pathway
UDP−N−acetyl−D−glucosamine biosynthesis
Semi−phosphorylative Entner−Doudoroff pathway
beta−Carotene biosynthesis
GABA
NAD
Lipoic acid biosynthesis
Nicotinate degradation
Aurachin biosynthesis
Dissimilatory nitrate reduction
Cytochrome aa3−600 menaquinol oxidase
Galactose degradation
Purine degradation
Complete nitrification
Denitrification
CMP−Neu5Ac biosynthesis
Glutathione biosynthesis
C4−dicarboxylic acid cycle
Catechol meta−cleavage
Propanoyl−CoA metabolism
ADP−L−glycero−D−manno−heptose biosynthesis
F−type ATPase
Cytochrome c oxidase
D−galactonate degradation
Ethylmalonyl pathway
Hydroxypropionate−hydroxybutylate cycle
Glycogen biosynthesis
Acetyl−CoA pathway
Reductive acetyl−CoA pathway
Ascorbate biosynthesis
Glucuronate pathway
2−Oxocarboxylic acid chain extension
Biotin biosynthesis
Tetrahydrofolate biosynthesis
Fatty acid biosynthesis
Fatty acid biosynthesis in mitochondria
beta−Oxidation
Methylaspartate cycle
3−Hydroxypropionate bi−cycle
Glycogen degradation
Bacillus anthracis pathogenicity signature
Assimilatory sulfate reduction
Nucleotide sugar biosynthesis
Phenylalanine biosynthesis
Menaquinone biosynthesis
Photorespiration
CAM
Glyoxylate cycle
Assimilatory nitrate reduction
Nitrate assimilation
Trehalose biosynthesis
Dissimilatory sulfate reduction
NADH:quinone oxidoreductase
Cysteine biosynthesis
Tyrosine biosynthesis
Adenine ribonucleotide degradation
Guanine ribonucleotide degradation
Pantothenate biosynthesis
Phosphatidylethanolamine
De novo pyrimidine biosynthesis
Molybdenum cofactor biosynthesis
Cyclooctatin biosynthesis
Thiamine biosynthesis
Polyamine biosynthesis
Tryptophan biosynthesis
Succinate dehydrogenase
Urea cycle
Heme biosynthesis
Pyridoxal−P biosynthesis
De novo purine biosynthesis
Siroheme biosynthesis
V/A−type ATPase
Arginine biosynthesis
Cobalamin biosynthesis
Adenine ribonucleotide biosynthesis
Methionine salvage pathway
Pentose phosphate pathway
PRPP biosynthesis
Pyrimidine ribonucleotide biosynthesis
Deoxyribonucleotide biosynthesis
Ectoine biosynthesis
Threonine biosynthesis
Ethylene biosynthesis
Mugineic acid biosynthesis
dTDP−L−rhamnose biosynthesis
Valine/isoleucine biosynthesis
Citrate cycle
Pyrimidine deoxyribonucleotide biosynthesis
L−threo−Tetrahydrobiopterin biosynthesis
Tetrahydrobiopterin biosynthesis
Histidine biosynthesis
Leucine degradation
Glycolysis
NAD biosynthesis
Pyruvate oxidation
C10−C20 isoprenoid biosynthesis
Shikimate pathway
Lysine biosynthesis
Gluconeogenesis
Reductive pentose phosphate cycle
Incomplete reductive citrate cycle
Serine biosynthesis
Ornithine biosynthesis
Reductive citrate cycle
C5 isoprenoid biosynthesis
Dicarboxylate−hydroxybutyrate cycle
Methionine biosynthesis
Lysine degradation
Pimeloyl−ACP biosynthesis
Methionine degradation
Nitrogen fixation
Formaldehyde assimilation
Inositol phosphate metabolism
Phenylacetate degradation
Coenzyme A biosynthesis
Ubiquinone biosynthesis
N−glycan precursor biosynthesis
F420 biosynthesis
Methanogenesis
Fumarate reductase
Methanofuran biosynthesis
Coenzyme M biosynthesis
Riboflavin biosynthesis
No Treatment
PMA
5000
4000
3000
2000
1000
