# Supplementary figures and images for: Comparison of Two DNA Labeling Dyes Commonly Used to Detect Metabolically Active Bacteria
Source: Microorganisms. 2025 Apr 28;13(5):1015. doi: 10.3390/microorganisms13051015 (PMC12114394; doi:10.3390/microorganisms13051015)

a.

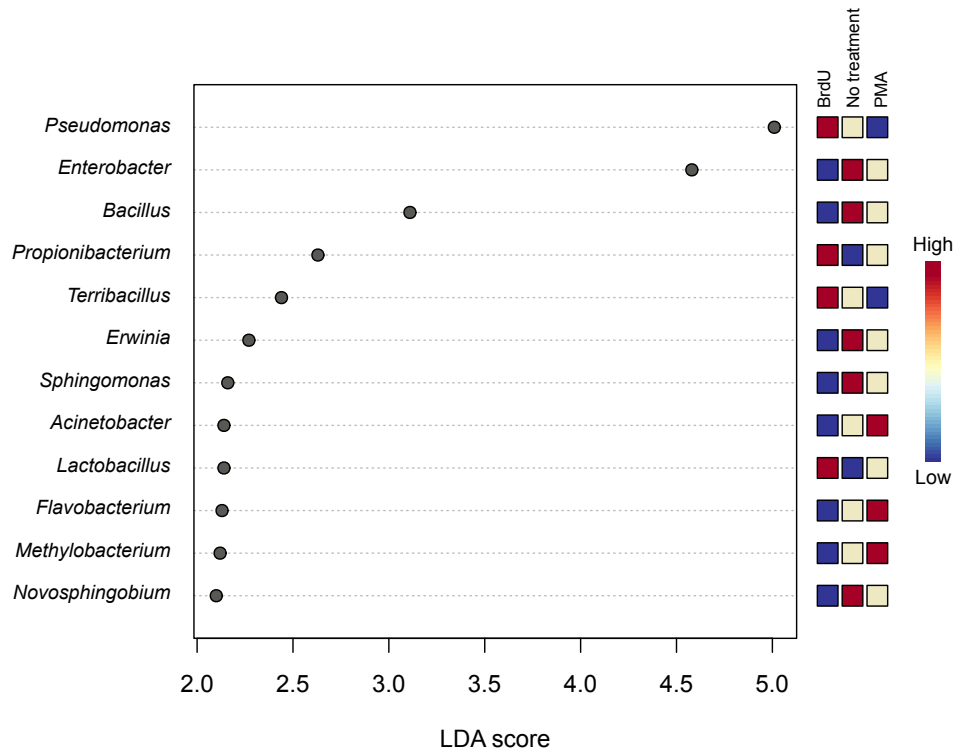

b.

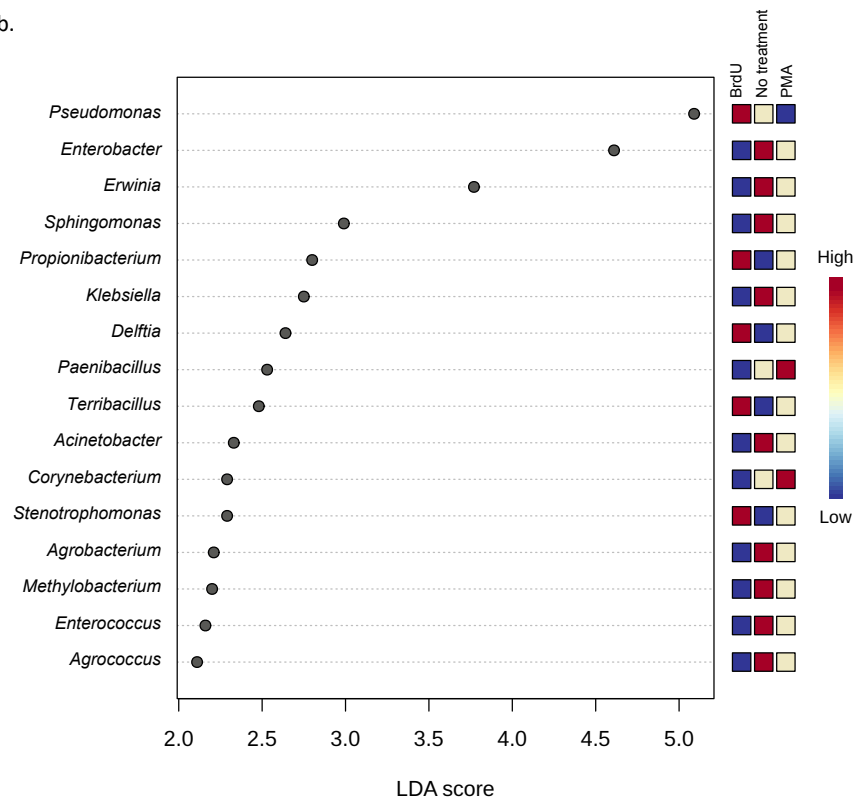

Supplement: Supplementary file 1 [file microorganisms-13-01015-s001.zip › Supplementary Figure S1.pdf]

a.

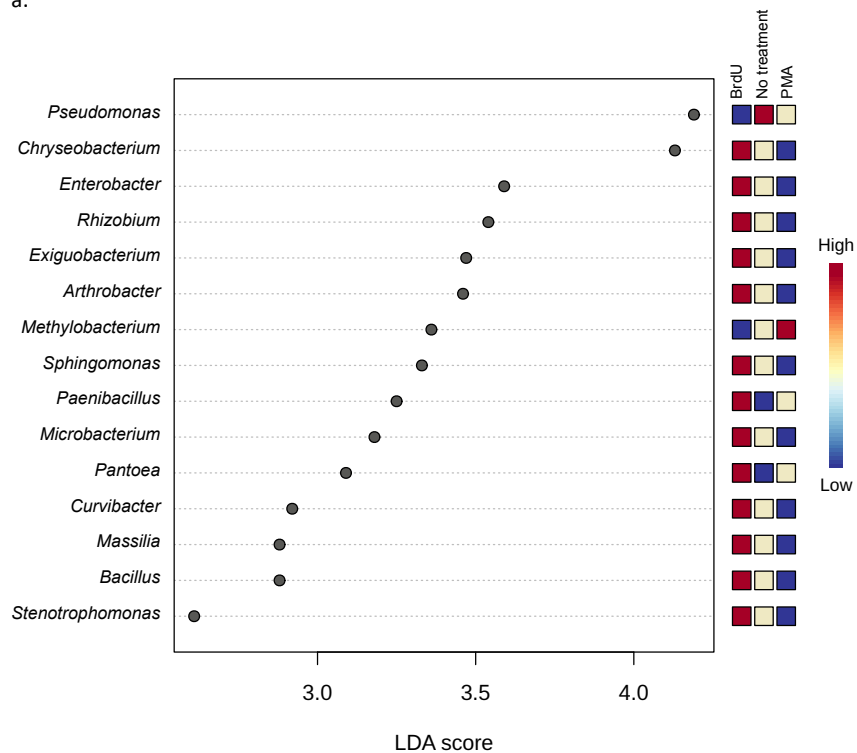

b.

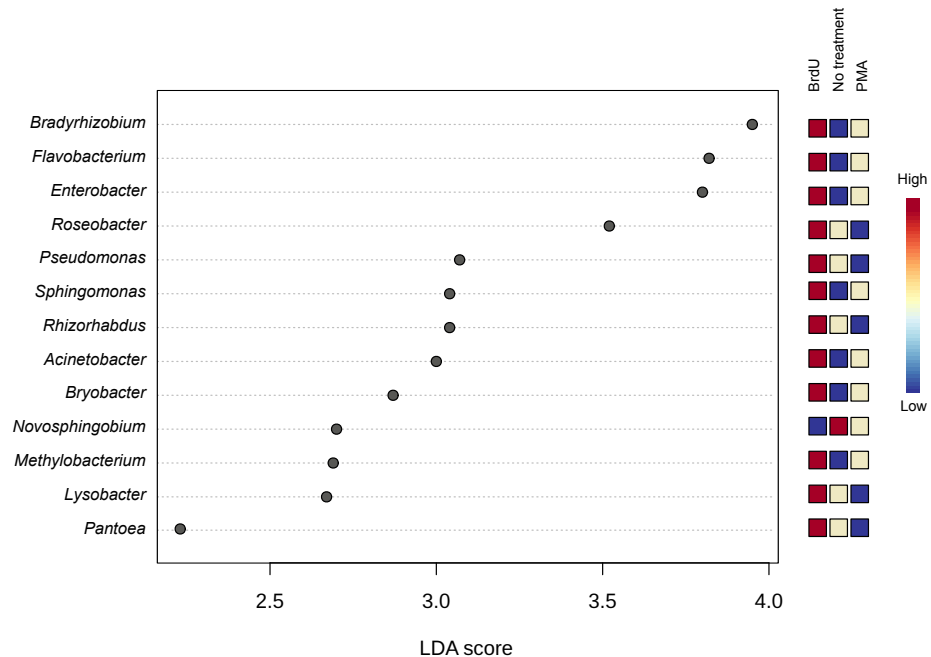

Supplement: Supplementary file 1 [file microorganisms-13-01015-s001.zip › Supplementary Figure S2.pdf]

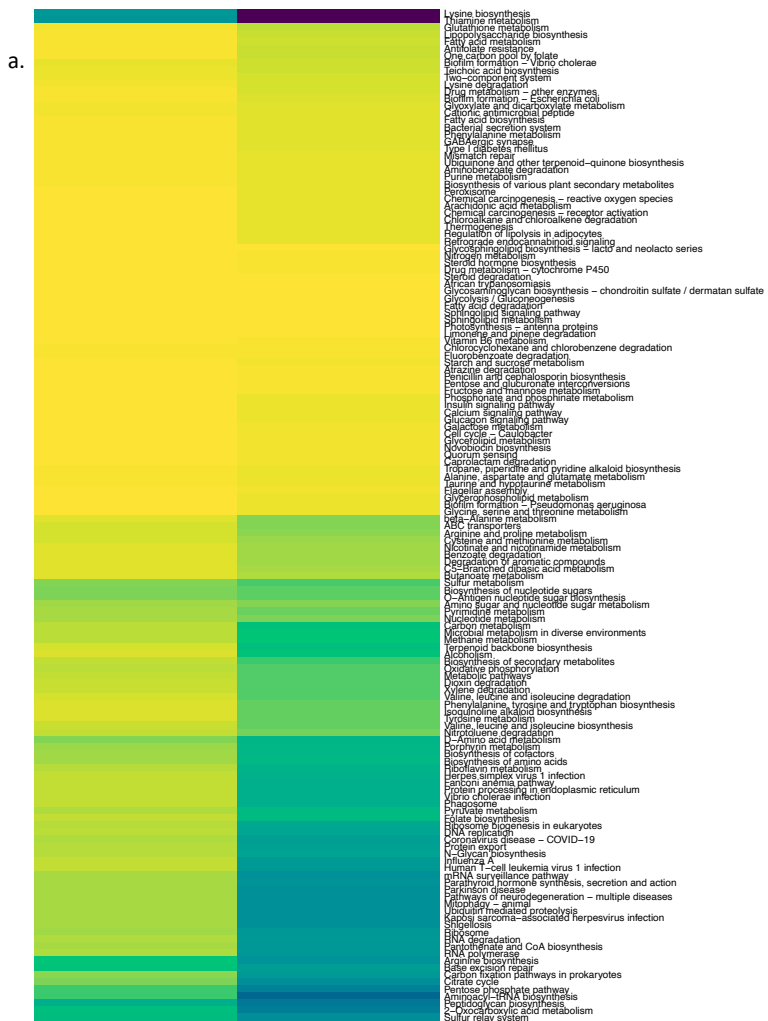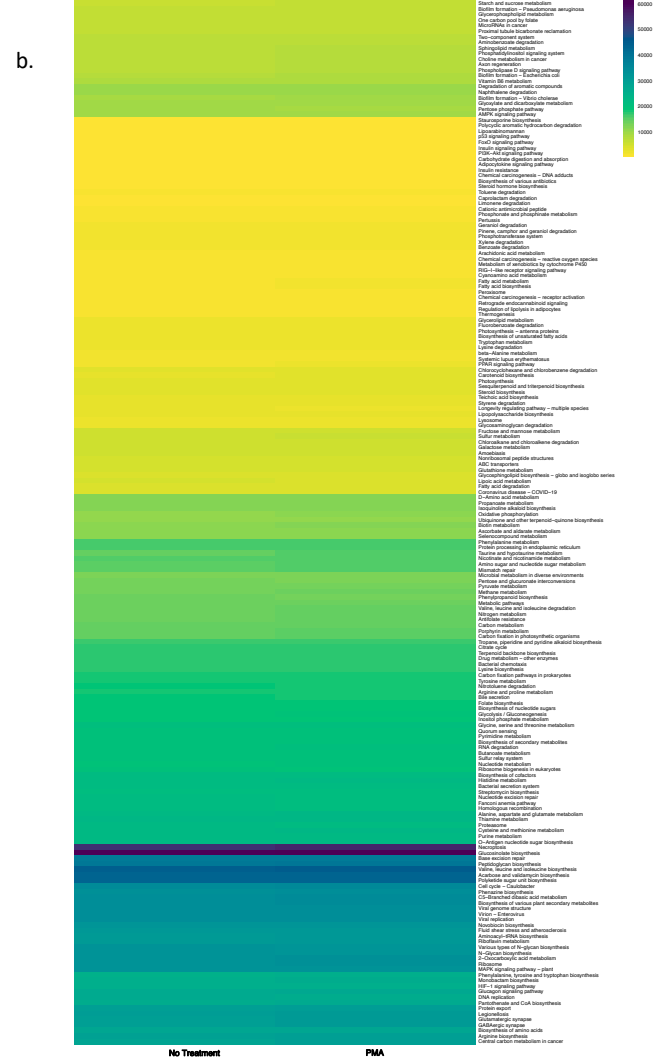

**BrdU**

### No Treatment

No Treatment

PMA

Supplement: Supplementary file 1 [file microorganisms-13-01015-s001.zip › Supplementary Figure S4.pdf]

a.

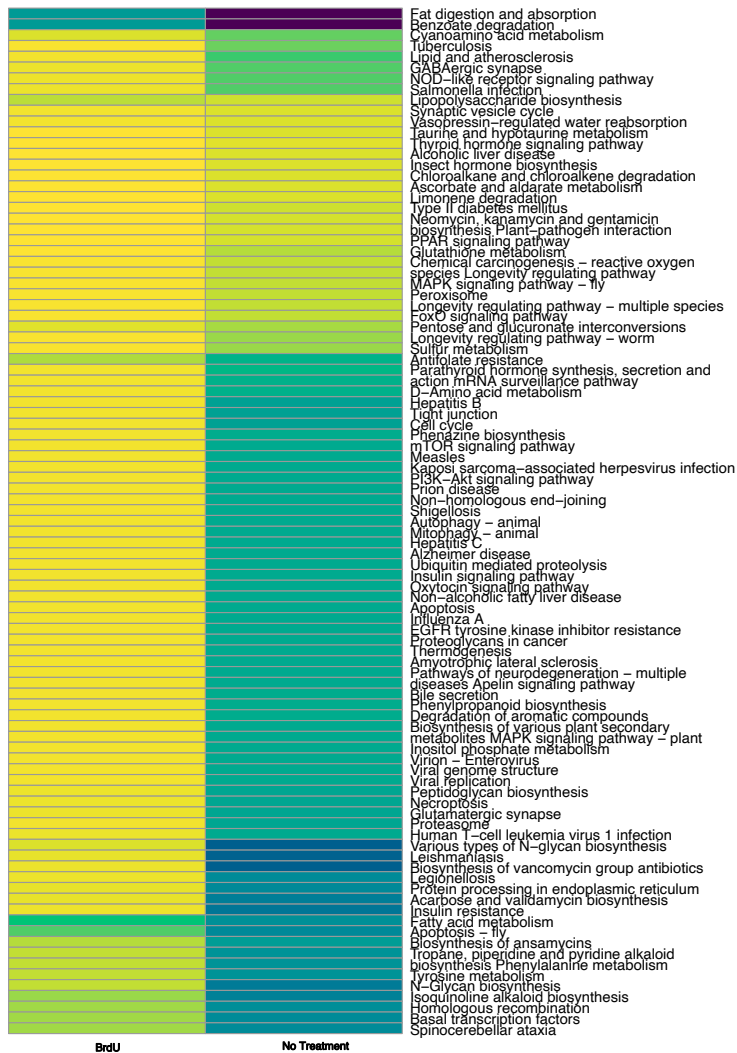

b.

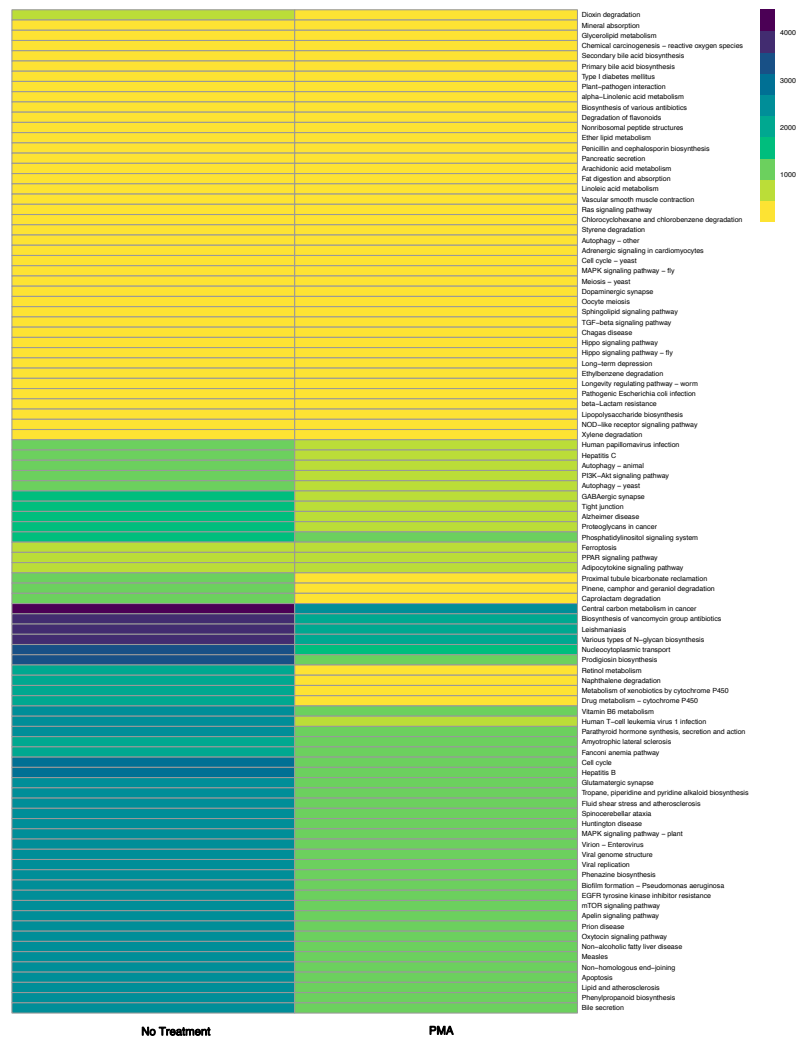

Supplement: Supplementary file 1 [file microorganisms-13-01015-s001.zip › Supplementary Figure S5.pdf]

a.

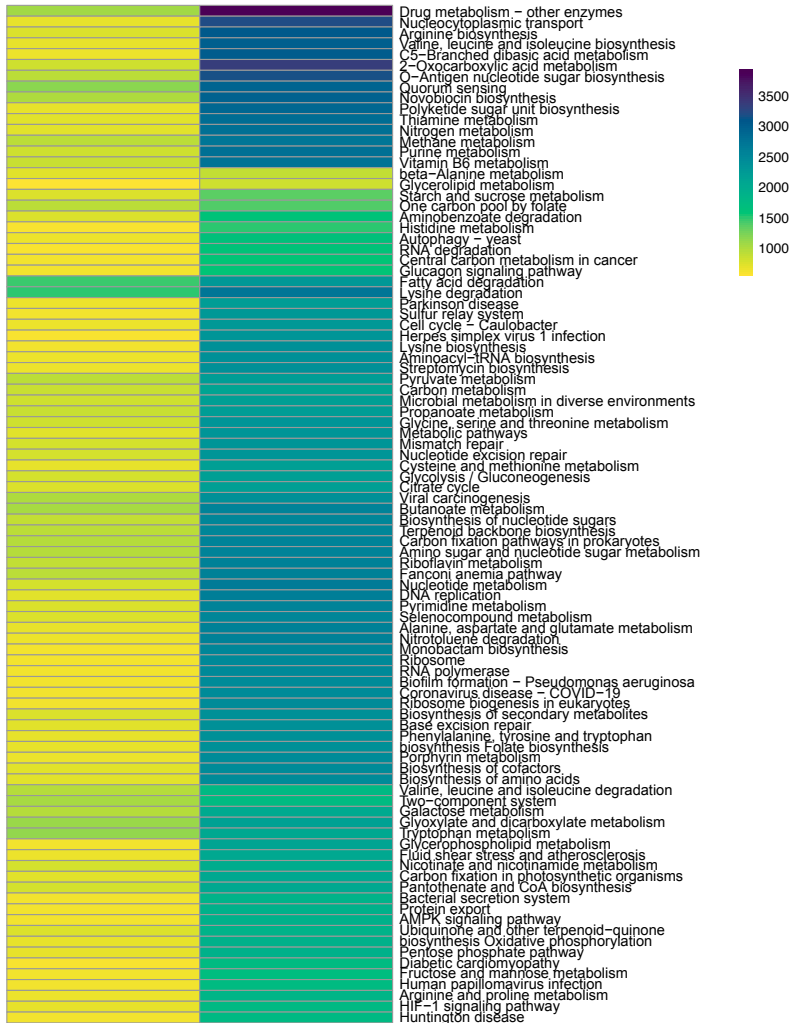

BrdU

No Treatment

b.

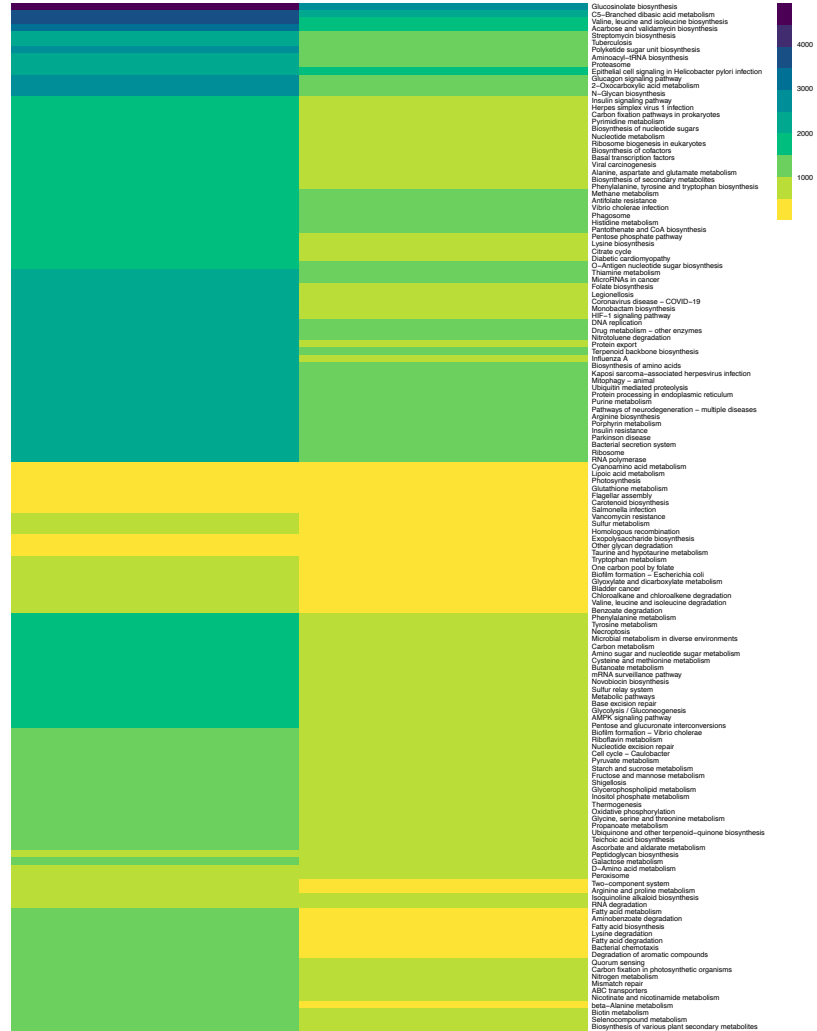

No Treatment

PMA

Supplement: Supplementary file 1 [file microorganisms-13-01015-s001.zip › Supplementary Figure S6.pdf]
